# Shikonin Prolongs Allograft Survival via Induction of CD4^+^FoxP3^+^ Regulatory T Cells

**DOI:** 10.3389/fimmu.2019.00652

**Published:** 2019-04-01

**Authors:** Qiaohuang Zeng, Feifei Qiu, Yuchao Chen, Cuihua Liu, Huazhen Liu, Chun-Ling Liang, Qunfang Zhang, Zhenhua Dai

**Affiliations:** Section of Immunology, The Second Clinical Medical College of Guangzhou University of Chinese Medicine, and Guangdong Provincial Academy of Chinese Medical Sciences, Guangzhou, China

**Keywords:** allograft rejection, immunosuppresant, immunoregulation, Treg, T cell, transplant immunology

## Abstract

A transplanted organ is usually rejected without any major immunosuppressive treatment because of vigorous alloimmune responsiveness. However, continuous global immunosuppression may cause severe side effects, including nephrotoxicity, tumors, and infections. Therefore, it is necessary to seek novel immunosuppressive agents, especially natural ingredients that may provide sufficient efficacy in immunosuppression with minimal side effects. Shikonin is a bioactive naphthoquinone pigment, an ingredient originally extracted from the root of *Lithospermum erythrorhizon*. Previous studies have shown that shikonin regulates immunity and exerts anti-inflammatory effects. In particular, it can ameliorate arthritis in animal models. However, it is unclear whether shikonin inhibits alloimmunity or allograft rejection. In this study and for the first time, we demonstrated that shikonin significantly prolonged the survival of skin allografts in wild-type mice. Shikonin increased the frequencies of CD4^+^Foxp3^+^ regulatory T cells (Tregs) post-transplantation and induced CD4^+^Foxp3^+^ Tregs *in vitro* as well. Importantly, depleting the Tregs abrogated the extension of skin allograft survival induced by shikonin. It also decreased the frequencies of CD8^+^CD44^high^CD62L^low^ effector T cells and CD11c^+^CD80^+^/CD11c^+^CD86^+^ mature DCs after transplantation. Moreover, we found that shikonin inhibited the proliferation of T cells *in vitro* and suppressed their mTOR signaling. It also reduced the gene expression of pro-inflammatory cytokines, including IFNγ, IL-6, TNFα, and IL-17A, while increasing the gene expression of anti-inflammatory mediators IL-10, TGF-β1, and indoleamine-2, 3-dioxygenase (IDO) in skin allografts. Further, shikonin downregulated IDO protein expression in skin allografts and DCs *in vitro*. Taken together, shikonin inhibits allograft rejection via upregulating CD4^+^Foxp3^+^ Tregs. Thus, shikonin is a novel immunosuppressant that could be potentially used in clinical transplantation.

## Introduction

Organ transplantation is an effective and final treatment for patients undergoing end-stage organ diseases (1, 2). However, almost all transplantation patients require continuous treatment with immunosuppressive drugs to prevent allograft rejection. Thus, immunosuppressive agents play an essential role in maintaining allograft survival. Conventional immunosuppressive drugs can effectively suppress allograft rejection and improve the outcome of transplantation through different mechanisms (3). However, global immunosuppression may also cause severe side effects. Adverse effects of an calcineurin inhibitor, such as cyclosporine (CsA), include nephrotoxicity, malignancies, hypertension, and infections (3–5) while rapamycin, an mTOR inhibitor, may result in hyperlipidemia, impaired glucose tolerance and diabetes, and acute renal toxicity (6, 7). Hence, it is necessary to search for novel immunosuppressive agents that may provide high efficiency in immunosuppression with minimal side effects.

Shikonin is a bioactive naphthoquinone pigment originally extracted from the root of *Lithospermum erythrorhizon* Sieb et Zucc. Shikonin has been utilized as an ingredient of the traditional Chinese herb to treat macular eruption, measles, sore throat, carbuncle, and burns in China for several centuries (8). Recent studies have also revealed that shikonin can exert anti-inflammatory, anticancer, and antimicrobial effects (9–11). In particular, shikonin has been demonstrated to inhibit the development of some immune-based inflammatory diseases, such as arthritis and asthma, in animal models (12, 13). Shikonin significantly decreased the severity of murine collagen-induced arthritis, alleviated the joint swelling and cartilage destruction. Further, it suppressed the production of matrix metalloproteinase (MMP)-1 and increased expression of tissue inhibitors of metalloproteinase (TIMP)-1 in this model (12). Shikonin suppressed allergic airway inflammation in a murine model of asthma by inhibiting the maturation of bone marrow-derived DCs (13). It also reduced IL-4, IL-5, IL-13, and TNF-α release in bronchial alveolar lavage fluid and lowered IL-4 and IL-5 production in lung cells and mediastinal lymph node cells. Importantly, shikonin has been proved to suppress human T lymphocyte activation via suppressing JNK signaling and IKKβ activity (14). Although shikonin has been shown to regulate immunity and inflammatory responses (10, 14, 15), it remains unknown whether shikonin modulates alloimmunity and suppresses allograft rejection. Here we hypothesized that shikonin could suppress alloimmune responses.

In current study, we found that shikonin significantly prolonged the survival of murine skin allografts. To our knowledge, this was the first evidence that shikonin inhibits allograft rejection in an experimental animal model. Shikonin significantly increased the frequencies of CD4^+^Foxp3^+^ regulatory T cell (Tregs) *in vivo* and induced CD4^+^Foxp3^+^ Tregs *in vitro* as well. Shikonin also decreased the frequencies of CD8^+^CD44^high^CD62L^low^ effector T cells and CD11c^+^CD80^+^/CD11c^+^CD86^+^ mature DCs after transplantation. Moreover, we demonstrated that shikonin inhibited the proliferation of T cells *in vitro* and suppressed their mTOR signaling. Finally, shikonin reduced the gene expression of proinflammatory cytokines in skin grafts while increasing IDO and FoxP3 protein expression in the grafts. Therefore, shikonin may represent a novel immunosuppressant that can be potentially applied to clinical transplantation.

## Materials and Methods

### Animals

C57BL/6 and BALB/c mice (6–8 week-old, weighing 20 ± 2 g) were purchased from Guangdong Medical Laboratory Animal Center (Guangzhou, China). All mice were housed in a specific pathogen-free room with controlled conditions. All experiments were approved by the Institutional Animal Ethical Committee of Guangdong Provincial Academy of Chinese Medical Sciences.

### Skin Transplantation

Skin donors were 6–8 week-old wild-type BALB/c mice while skin graft recipients were 6–8 week-old C57BL/6 mice. Full-thickness trunk skin with an approximate size of 10 mm^2^ was transplanted to the dorsal flank area of recipient mice and secured with a bandage of Band-Aid (Johnson Johnson, New Brunswick, NJ). The bandage was removed 8 days after transplantation. Skin allograft rejection was monitored daily and defined as graft necrosis >90%, as also described in our previous publication (16).

### Administration of Drugs

Mice were randomly grouped into control groups, and experimental groups that were administrated with shikonin (*p.o*. 20 or 40 mg/kg body weight) or cyclosporine (CsA, *i.p*. 20 mg/kg, or 10 mg/kg for joint treatment with shikonin) for a period of 2 consecutive weeks or until graft rejection/sample collection. Anti-CD45RB Ab (Clone MB23G2: Bio X Cell, NH, USA) was injected *i.p*. at 0.1 mg/day on days 0 and 5. CsA (Sigma, USA) was dissolved in saline while shikonin (Push Bio-technology, China) was prepared with sodium carboxymethyl cellulose (CMC-Na; Sigma). Control groups were administrated orally with equivalent CMC-Na solution only. Our previous study has shown that oral administration of CMC-Na (vehicle) did not alter allograft rejection compared with untreated groups (unpublished observation). To deplete Tregs, recipient mice were administrated *i.p*. with anti-CD25 Ab (eBioscience, USA) at 0.2 mg on days 0, 4, and 8 post-transplantation as described in our previous publication (17).

### Histological Analysis

Skin grafts of recipient mice were harvested, fixed with 4% paraformaldehyde for more than 24 h and then embedded in paraffin. The sections (3 μm) of skin tissues were then made and stained with Hematoxylin and Eosin (H&E). In addition, sections were incubated first with primary anti-CD3 antibody (1:100, Abcam, USA) at 4°C overnight, then with secondary antibody HRP-anti-Rabbit IgG (Maxim, China) at 37°C for 30 min, and finally colored with diaminobenzidine at room temperature without light for immunohistochemical analysis.

### Immunofluorescence

Skin grafts of recipient mice were harvested, embedded in OTC and frozen. Skin tissue sections were cut with a thickness of 3 μm using freezing microtome. Then they were incubated in 0.3% Triton X-100 and 10% bovine serum albumin for 1 h, following by incubation overnight at 4°C with primary mouse anti-indoleamine-2, 3-dioxygenase (Millipore, USA) and rabbit anti-CD11c (Cell Signaling Technology, USA) or rabbit anti-Foxp3 (Cell Signaling Technology) antibody at a concentration of 1:100. Sections were then stained with a secondary antibody Alexa Fluor® 555-conjugated anti-mouse IgG or Alexa Fluor® 488 conjugated anti-rabbit IgG (Cell Signaling Technology). These cryosections were finally mounted using DAPI-Fluoromount-G clear mounting agents (SouthernBiotech, Birmingham, UK). All of the images were obtained randomly using a fluorescence microscope (magnification 200×).

### Flow Cytometric Analysis

Draining lymph node (LN) and spleen cells were harvested and stained with anti-CD4-PE (Clone H129.19), CD8-FITC (Clone 53-6.7), CD44-V450 (Clone IM7), CD11c-PE (Clone HL3), CD80-FITC (Clone 16-10A1), CD86-FITC (Clone GL1), and anti-CD62L-APC (Clone MEL-14) antibodies (all from BD Biosciences, USA) to analyze surface markers. Intracellular FoxP3 staining was performed using Foxp3/Transcription Factor Fixation/Permeabilization Concentrate and Diluent Kit (eBioscience, USA) according to manufacturer's instruction. Then, some cells were stained with anti-FoxP3-APC antibody (Clone FJK-16s, eBioscience). The stained cells were washed and finally analyzed using a flow cytometer (FACSCalibur, BD Biosciences). To isolate CD3^+^ T cells and CD4^+^CD25^−^ T cells, spleen cells were isolated and stained with anti-CD3-APC (Clone 145-2C11), CD4-FITC (Clone H129.19), and anti-CD25-PE (Clone 3C7) Abs (BD Biosciences). CD3^+^ T cells and CD4^+^CD25^−^ T cells were then purified using FACSAria III cell sorter (BD Biosciences). The purity of the sorted cells was typically >96%.

### Induction of Tregs *in vitro*

CD4^+^CD25^−^ T cells from C57BL/6 mice were sorted out using FACSAria III cell sorter. Then, cells (4 × 10^5^ cells/well) were cultured in 96-well plates (200 μl/well) in complete RPMI-1640 medium (Gibico, USA) containing 10% fetal bovine serum (Gibico), 100 U/ml penicillin and 100 μg/ml streptomycin, and stimulated with anti-CD3/anti-CD28 Abs (2.5 μg/ml) and IL-2 (10 ng/ml, Peprotech) in the absence or presence of TGF-β1 (positive control, 5 ng/ml, Peprotech) or shikonin (0.25 or 0.5 μM) for 4 days. The frequency of CD4^+^Foxp3^+^ Tregs was finally determined using a FACSCalibur.

### T Cell Proliferation Assay and Measurement of Cytokines *in vitro*

FACS-sorted CD3^+^ T cells derived from C57BL/6 mice were labeled with 3 μM CFSE (Invitrogen, Germany) at room temperature without light for 15 min. Then, cells (4 × 10^5^ cells/well) were cultured in 96-well plates in complete RPMI-1640 medium as described above and stimulated with anti-CD3/anti-CD28 Abs (2.5 μg/ml) plus IL-2 (10 ng/ml, Peprotech) at 37°C with 5% CO_2_ for 4 days. These cells were also treated with either shikonin (0.25 or 0.5 μM) or CsA (0.1 μM) for 4 days. Finally, cell proliferation was measured using a FACSCalibur. On the other hand, the levels of IFN-γ, IL-10, TGF-β1, and IL-17A in the supernatant were also measured using ELISA according to the manufacturer's instructions (Boster, China), and the absorbance was read at 450 nm in a microplate spectrophotometer (Thermo Fisher Scientific, USA).

### Assays of T Cell Cytotoxicity *in vitro*

T cell cytotoxicity was detected using CCK-8 assays. FACS-sorted CD3^+^ T cells derived from C57BL/6 mice were cultured in 96-well plates in complete RPMI-1640 medium (Gibico, USA) containing 10% fetal bovine serum (Gibico), 100 U/ml penicillin and 100 μg/ml streptomycin, and stimulated with anti-CD3/anti-CD28 Abs (2.5 μg/ml) and IL-2 (10 ng/ml, Peprotech). Shikonin was added to each well at different concentrations (0.1, 0.25, 0.5, 1, and 2 μM, respectively) with four wells *per* concentration. After 24, 48, and 96 h, 20 μL of CCK-8 was added to each well and incubated at 37°C for 4 h. The absorbance was measured by a microplate spectrophotometer (Thermo Fisher Scientific, USA) at the wavelength of 450 nm. Control without shikonin was set as 1.0.

### Quantitative Real-Time Reverse Transcription PCR (qRT-PCR)

Total mRNA from a skin graft was isolated using Trizol reagents (Invitrogen, USA) and mRNA was then transcribed to cDNA using a PrimeScript™ RT reagent kit (Takara Bio Incorporation, Kusatsu, Japan) according to the instructions of the manufacturer. The cDNA was analyzed for the expression of cytokines using a Quantifast SYBR Green PCR kit (Takara Bio Incorporation) via an ABI 7500 Fast RealTime PCR System (Thermo Fisher Scientific). The primer sequences were shown in Table 1. The relative mRNA expression levels of cytokines were normalized against β-actin, and analysis was performed through a comparative 2^ΔΔCT^ method. All data are shown in the form of relative expression as fold changes.

**Table 1:** Primer sequences of target genes.

**Target gene**	**Primer sequence (5′ → 3′)**
IFN-**γ** (forward)	CACGGCACAGTCATTGAAAG
IFN-**γ** (reverse)	CATCCTTTTGCCAGTTCCTC
TNF-α (forward)	ACGGCATGGATCTCAAAGAC
TNF-α (reverse)	GTGGGTGAGGAGCACGTAGT
IL-10 (forward)	CCAAGCCTTATCGGAAATGA
IL-10 (reverse)	TCCTGAGGGTCTTCAGCTTC
IL-17A (forward)	GTCCAAACACTGAGGCCAAG
IL-17A (reverse)	ACGTGGAACGGTTGAGGTAG
IL-6 (forward)	ACTTCCATCCAGTTGCCTTCTTGG
IL-6 (reverse)	TTAAGCCTCCGACTTGTGAAGTGG
Foxp3 (forward)	CCCATCCCCAGGAGTCTTG
Foxp3 (reverse)	ACCATGACTAGGGGCACTGTA
IDO (forward)	GCTTTGCTCTACCACATCCAC
IDO (reverse)	CAGGCGCTGTAACCTGTGT
TGF-β1 (forward)	CAATTCCTGGCGTTACCTTG
TGF-β1 (reverse)	AGCCCTGTATTCCGTCTCCT
β-actin (forward)	TGTCCACCTTCCAGCAGATGT
β-actin (reverse)	TGTCCACCTTCCAGCAGATGT

### Generation of Dendritic Cells *in vitro* From Bone Marrow

Bone marrow cells were harvested from femurs of C57BL/6 mice. Cells (1 × 10^6^ /mL) were cultured overnight in 12-well plates with complete RPMI-1640 medium in the presence of recombinant mouse rGM-CSF (20 ng/ml, Peprotech) plus rIL-4 (10 ng/ml, Peprotech). Adherent cells were further incubated with the complete medium containing fresh rGM-CSF and rIL-4 for 5 days before suspension cells were removed. Finally, these dendritic cells (DCs) were treated with either shikonin (0.25 or 0.5 μM) or CsA (0.1 μM) for 48 h. Total protein samples from DCs were obtained and then measured for IDO protein expression.

### Western Blotting

Total protein samples from T cells or DCs were obtained in RIPA lysis buffer followed by centrifugation (12,000 rpm and 10 min) in 4°C. Then, the protein concentration was measured using a BCA protein assay kit (Thermo Fisher Scientific). Samples were run on 10% SDS-PAGE gels and electro-transferred onto a PVDF membrane. TBST with 5% (w/v) BSA was used to block non-specific binding to the membrane at room temperature for 1 h. The membrane was then incubated with a primary antibody anti-phospho-P70S6K, anti-P70S6K, anti-phospho-mTOR, anti-mTOR (1:1,000; Cell Signaling Technology, USA), or anti-IDO (1:1,000; Millipore, USA) at 4°C overnight. After incubation, the membrane was washed and incubated with a secondary antibody, HRP-conjugated goat anti-rabbit IgG (1:2,000) for 1 h. GAPDH (1:2,000; Cell Signaling Technology) was also used for loading controls. Finally, blots were visualized by an ECL method (Beyotime, China) and analyzed using an Image J Program software.

### Statistical Analysis

Comparisons of means were conducted using Student's *t*-test and one-way ANOVA. Data were presented as the mean ± SD and analyzed through GraphPad Prism 6 (GraphPad Software, USA). The analysis of graft survival was performed using Kaplan–Meier method (log-rank test). A value of *P* < 0.05 was considered statistically significant.

## Results

### Shikonin Significantly Prolongs Skin Allograft Survival and Reduces CD3^+^ T Cell Infiltration in the Skin Allografts

**Figure 1:**
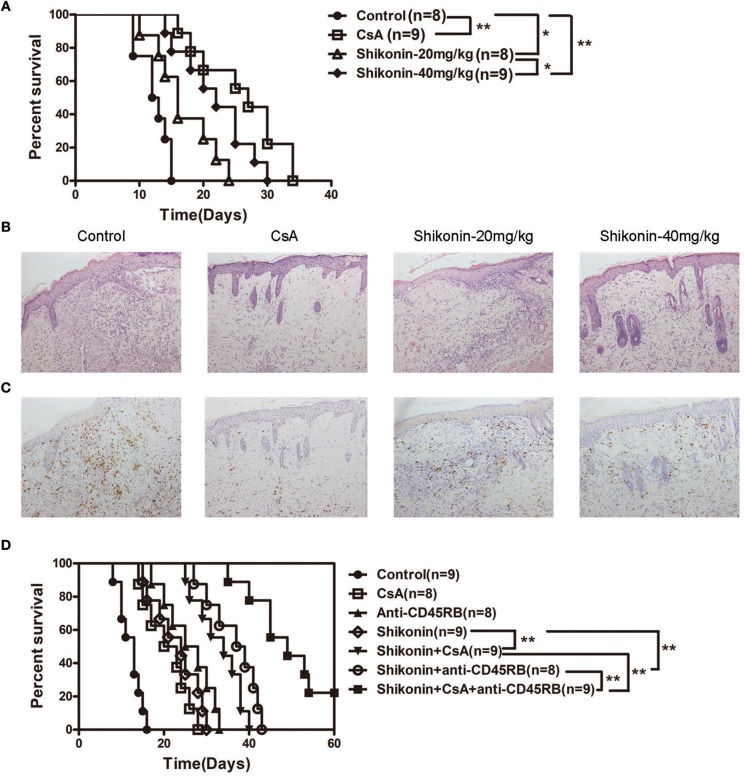
Shikonin prolongs skin allograft survival and reduces CD3^+^ T cell infiltration in skin allografts. **(A)** C57BL/6 mice were transplanted with a skin allograft from a BALB/C donor and treated without or with shikonin or CsA (20 mg/kg). The survival times of the skin grafts were observed (*P < 0.05 and **P < 0.01, *n* = 8–9). H&E **(B)** and immunohistochemical staining for CD3 **(C)** were performed 10 days post-transplantation. **(D)** Transplanted mice were treated with shikonin plus low doses of CsA (10 mg/kg) and/or anti-CD45RA mAb (0.1 mg) to observe the effects of the double or triple treatments on skin allograft survival (**P < 0.01, *n* = 8–9).

To investigate whether shikonin would suppress allograft rejection, we used BALB/C mice as skin donors and C57BL/6 mice as skin recipients that were then treated with shikonin or CsA for 14 consecutive days following transplantation. As shown in Figure 1A, we found that both low and high doses of shikonin significantly prolonged skin allograft survival compared to the control group [median survival time (MST) = 16 (low dose) vs. 12 days and 22 (high dose) vs. 12 days, both *P* < 0.05]. Interestingly, treatment with high doses of shikonin was nearly as effective as CsA (20 mg/kg) in prolongation of skin allograft survival (MST = 22 vs. 26 days, *P* > 0.05). On the other hand, the skin allografts were analyzed via H&E or immunohistochemical (IHC) staining 10 days post-transplantation. We found that treatment with either shikonin or CsA obviously reduced overall cellular infiltration (Figure 1B) as well as CD3^+^ T-cell infiltration (Figure 1C) in the skin allografts compared to the control group, suggesting that shikonin prolongs skin allograft survival and reduces CD3^+^ T-cell infiltration in the allografts. Moreover, we examined the effects of combined treatments with shikonin and low doses of CsA (10 mg/kg) or anti-CD45RB Ab (0.1 mg at day 0 & 5), and found that the double treatment was more effective in suppression of allograft rejection than shikonin alone while the triple treatment with all of three agents further prolonged allograft survival compared to the double treatment with either Shikonin+CsA or Shikonin+anti-CD45RB Ab (Figure 1D). However, most of the allografts (7/9) in recipient mice with the triple treatment were still rejected within 60 days.

### Shikonin Induces CD4^+^ Foxp3^+^ Tregs *in vivo and in vitro*

**Figure 2:**
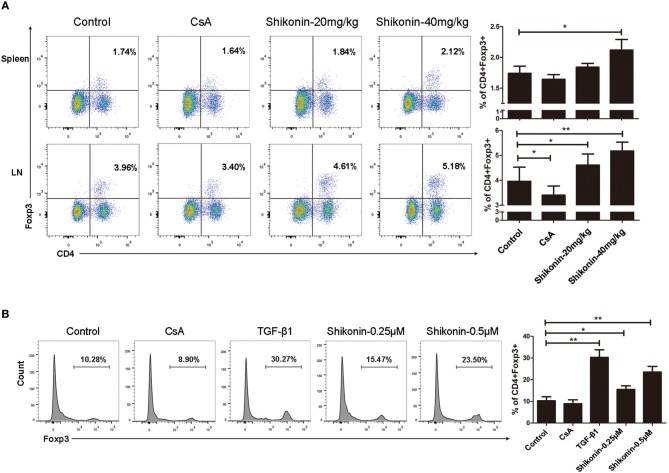
Shikonin induces CD4^+^Foxp3^+^ Tregs *in vivo and in vitro*. **(A)** Draining lymph node and spleen cells were isolated from C57BL/6 mice that were transplanted with skin allografts from BALB/C mice and treated with shikonin or CsA for 8 consecutive days. CD4^+^Foxp3^+^ Tregs were then enumerated via FACS analysis. **(B)** FACS-sorted CD4^+^CD25^−^ T cells from C57BL/6 mice were co-stimulated with anti-CD3/CD28 and IL-2 in the absence or presence of TGF-β1 or shikonin for 4 days. The frequencies of CD4^+^Foxp3^+^ Tregs were determined using a flow cytometer. Data of column graphs are presented as means ± SD (*P < 0.05 and **P < 0.01, *n* = 4–5 mice or wells). One of three separate experiments is shown.

Regulatory T cells play a key role in maintaining immune homeostasis and tolerance (18–20). Thus, we examined whether shikonin would prolong skin allograft survival by inducing CD4^+^Foxp3^+^ Tregs *in vivo and in vitro*. Draining lymph node and spleen cells were isolated 8 days after transplantation, and CD4^+^Foxp3^+^ Tregs were detected using flow cytometric analysis. As shown in Figure 2A, shikonin significantly increased the frequencies of CD4^+^Foxp3^+^ Tregs in draining lymph nodes compared with the control group while CsA did the opposite. Moreover, high doses of shikonin increased the Treg frequencies more drastically than did its low doses. In contrast, shikonin at high doses, but not low doses, significantly augmented the frequencies of splenic Tregs post-transplantation. Shikonin also increased the Treg frequencies in recipient mice 14 days after skin transplantation (data not shown). Since shikonin increased CD4^+^Foxp3^+^ Tregs in recipient mice, we asked if shikonin would also promote the induction of CD4^+^Foxp3^+^ Tregs *in vitro*. FACS-sorted CD4^+^CD25^−^ T cells from C57BL/6 mice were activated by anti-CD3/CD28 Abs in the presence of IL-2 without or with TGF-β1 or shikonin for 4 days, and CD4^+^Foxp3^+^ Tregs were then quantified via FACS analysis. As shown in Figure 2B, shikonin, at both low and high concentrations, significantly increased the percentages of Foxp3^+^ Tregs *in vitro*, and so did TGFβ1, a positive control. However, CsA did not augment the Treg frequency. Taken together, our data suggest that shikonin promotes CD4^+^Foxp3^+^ Treg generation both *in vivo* and *in vitro*.

### Shikonin Increases Both IDO and FoxP3 Expressions in Skin Allografts While Augmenting IDO Expression by DCs *in vitro*

**Figure 3:**
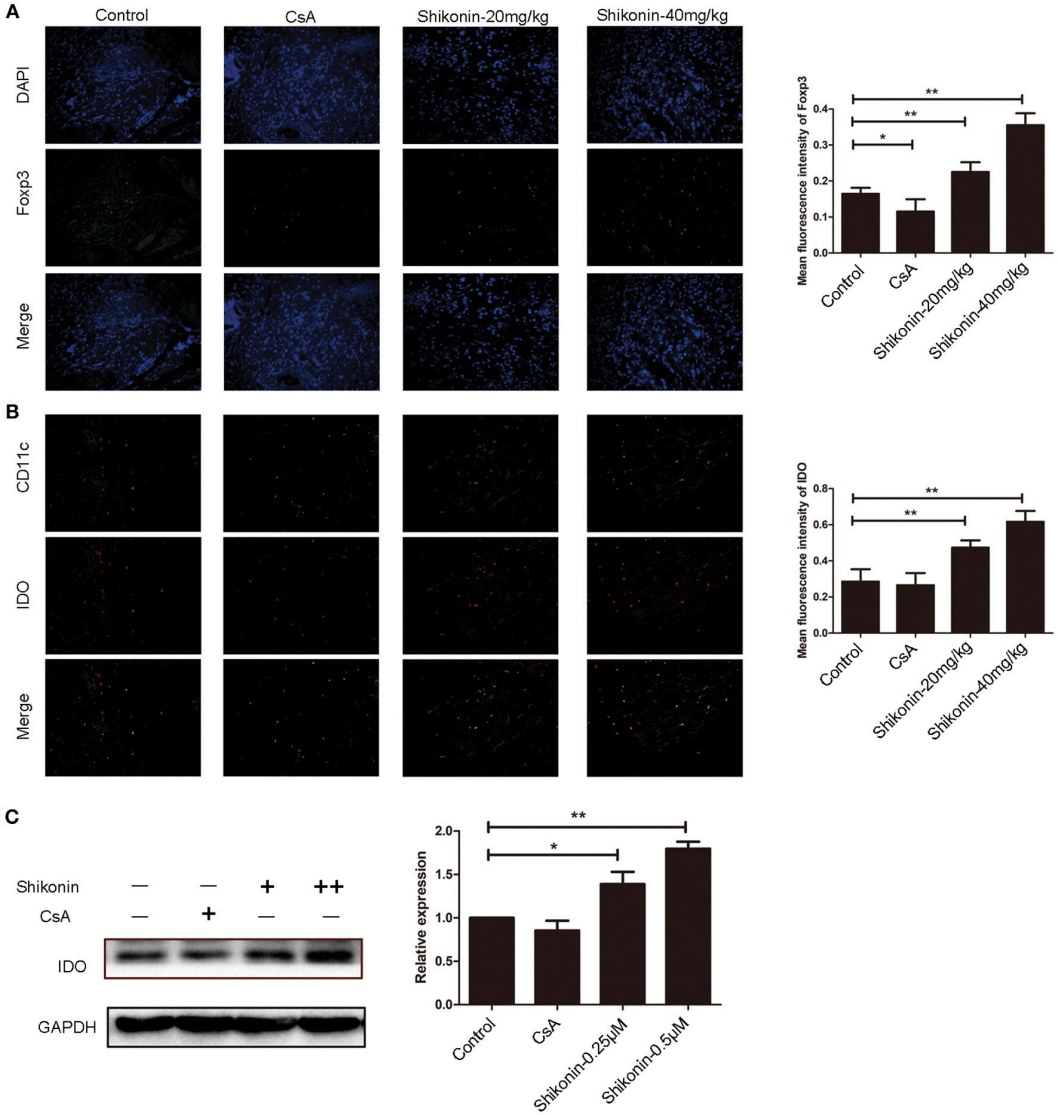
Shikonin increases IDO expression in skin allografts and DCs *in vitro*. Immunofluorescence staining of FoxP3 **(A)** and IDO **(B)** on skin allografts was performed 10 days following skin allotransplantation. Merged images from double stainings of CD11c/IDO or DAPI/FoxP3 are also shown. Data of column graphs on right panels are presented as means ± SD of mean fluorescence intensity from three separate experiments (**P < 0.01). On the other hand, bone marrow-derived DCs were cultured in the absence or presence of shikonin for 2 days. IDO protein expression by DCs was then determined via Western blot **(C)**. One of three sets of images is shown. Data of column graphs are presented as means ± SD relative to control from three separate experiments (*P < 0.05 and **P < 0.01).

It is generally accepted that IDO can induce CD4^+^Foxp3^+^ Tregs. We then asked whether shikonin would increase IDO protein expression in skin allografts. Immunofluorescence staining of IDO and FoxP3 on skin allografts was performed 10 days following skin transplantation. As shown in Figure 3A, shikonin, but not CsA, augmented FoxP3 expression in skin allografts. Furthermore, it also increased IDO expression in the grafts while IDO was mainly expressed by DCs, as demonstrated by the double stainings of both IDO and CD11c (Figure 3B). On the other hand, bone marrow-derived DCs were cultured in the absence or presence of shikonin for additional 2 days. IDO protein expression by DCs was then determined via Western blot analysis. As shown in Figure 3C, it was shikonin, but not CsA, that enhanced IDO protein expression in DCs *in vitro*, suggesting that shikonin upregulates IDO protein expression *in vivo* and *in vitro*.

### Shikonin Hinders DC Maturation Post-Transplantation

**Figure 4:**
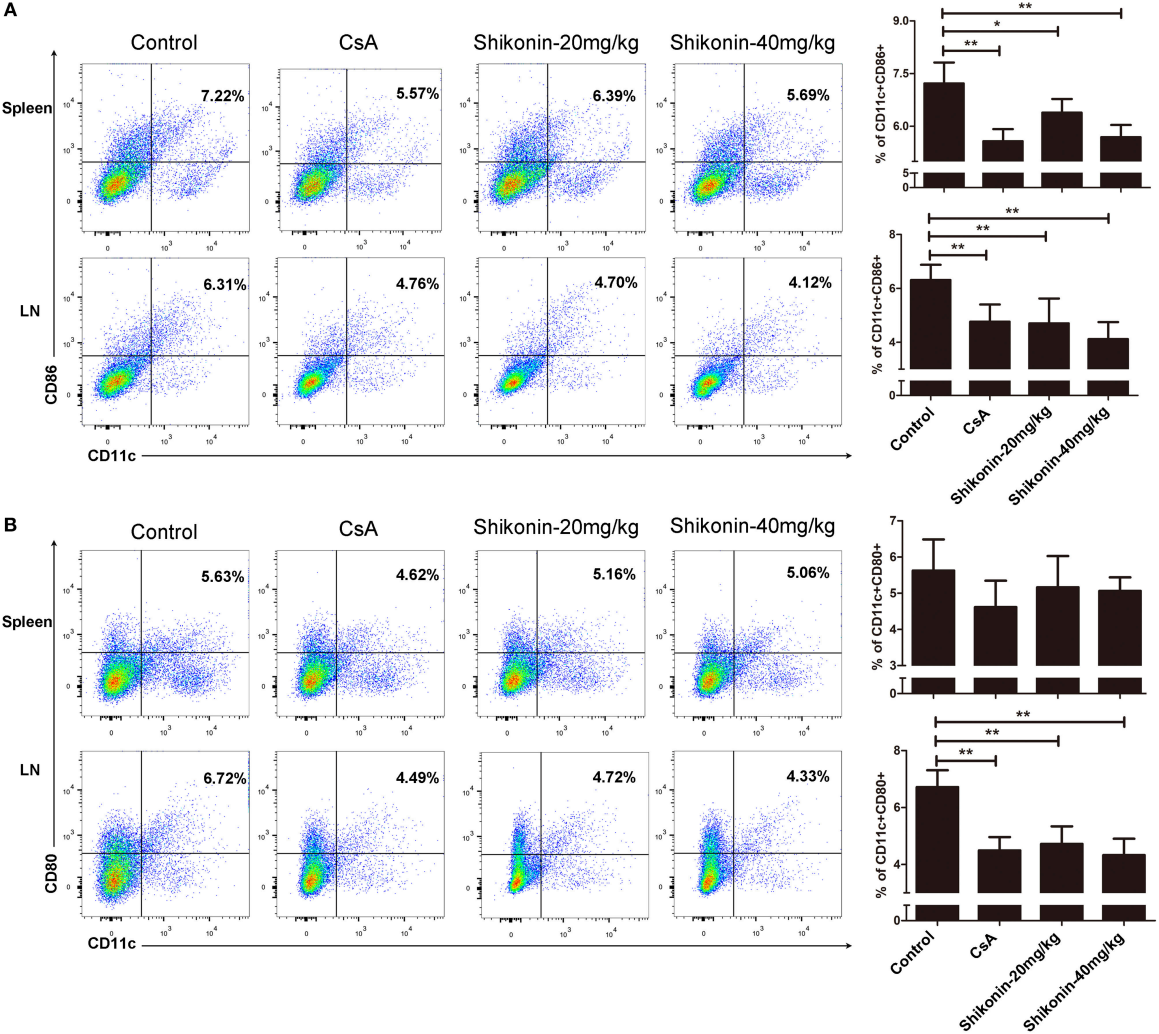
Shikonin hinders DC maturation in allotransplantation. Splenocytes and lymph node cells were isolated from recipient mice and analyzed for the markers of DC maturation 10 days after skin transplantation and treatment with CsA or shikonin. The percentages of CD11c^+^CD86^+^ **(A)** or CD11c^+^CD80^+^ **(B)** mature DCs were determined by FACS analysis. Data of column graphs are presented as means ± SD from three separate experiments (*P < 0.05 and **P < 0.01).

To determine if shikonin would also have an impact on DC maturation in the context of allotransplantation, splenocytes, and lymph node cells were analyzed for the markers of DC maturation 10 days after skin transplantation. As shown in Figure 4A, either CsA or shikonin significantly reduced the percentage of CD11c^+^CD86^+^ mature DCs in both the spleen and lymph nodes. As for CD11c^+^CD80^+^ cells, another subset of mature DCs, shikonin only decreased their frequency in the draining lymph nodes, but not the spleen (Figure 4B). These findings indicate that shikonin does interfere with DC maturation.

### Depletion of CD25^+^ Tregs Reverses Allograft Survival Prolonged by Shikonin

**Figure 5:**
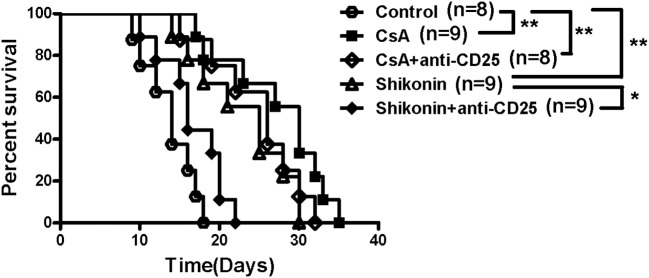
Depleting CD25^+^ Tregs reverses skin allograft survival prolonged by shikonin. C57BL/6 mice were transplanted with BALB/C skin and treated with shikonin (40 mg/kg) or CsA (20 mg/kg) for 14 consecutive days or until allograft rejection. They were also depleted of CD4^+^CD25^+^ Tregs through injection of anti-CD25 Ab (PC61), as described in the methods. Skin allograft survival times were observed (*P < 0.05 and **P < 0.01, *n* = 8–9 mice).

To investigate whether the effects of shikonin on allograft survival were dependent on its induction of CD4^+^CD25^+^ Tregs, C57BL/6 mice transplanted with BALB/C skin were treated with shikonin (40 mg/kg). They were then depleted of CD25^+^ Tregs through injection of anti-CD25 mAb (PC61). As shown in Figure 5, we found that depleting CD25^+^ Tregs mostly reversed skin allograft survival prolonged by shikonin but not CsA. Isotype control Ab did not reverse it (our unpublished observation). These findings suggest that extension of allograft survival by shikonin is largely dependent on its induction of CD4^+^CD25^+^ Tregs.

### Shikonin Reduces Effector CD8^+^ T Cell Frequency *in vivo*

**Figure 6:**
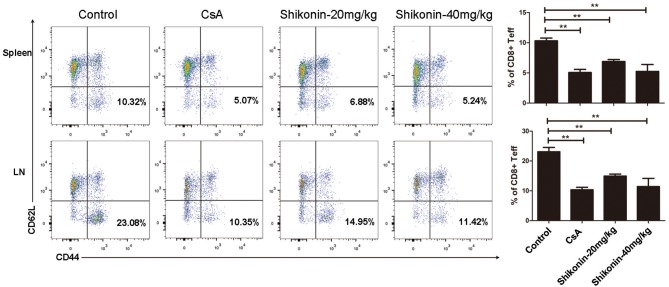
Shikonin decreases effector CD8^+^ T cell frequency *in vivo*. C57BL/6 mice were transplanted with BALB/C skin and treated with shikonin or CsA daily. Draining lymph node and spleen cells were isolated from the recipient mice 10 days after transplantation, and the percentages of CD8^+^CD44^high^CD62L^low^ effector T cells (Teff) were determined using a flow cytometer. Data of column graphs are presented as means ± SD (**P < 0.01, *n* = 4–6 mice). One representative of three separate experiments is shown.

To determine whether shikonin would suppress effector CD8^+^ T cells *in vivo*, draining lymph node and spleen cells were isolated from C57BL/6 recipients treated with shikonin or CsA. The cells were then analyzed for the frequency of CD8^+^CD44^high^CD62L^low^ effector T cells (Teff) through a flow cytometer 10 days after transplantation. As represented by Figure 6, shikonin, at either low or high doses, significantly reduced the percentage of CD8^+^CD44^high^CD62L^low^ effector T cells in both lymph nodes and spleens of the recipients compared with the control group. As a control, CsA also did the same. These findings suggest that shikonin suppresses the expansion of CD8^+^CD44^high^CD62L^low^ effector T cells in transplanted recipient mice.

### Shikonin Suppresses T cell Proliferation and Production of Proinflammatory Cytokines *in vitro*

**Figure 7:**
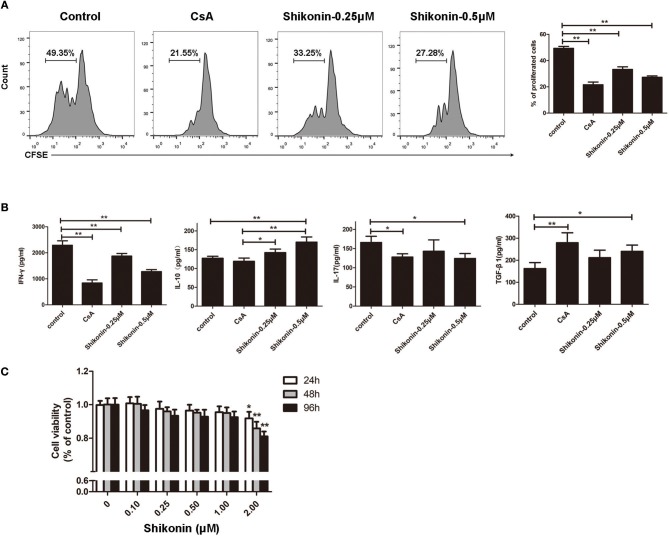
Shikonin suppresses T cell proliferation and production of proinflammatory cytokines *in vitro*. **(A)** FACS-sorted CD3^+^ T cells from C57BL/6 mice were labeled with CFSE and then co-stimulated with anti-CD3/CD28 Abs and IL-2 in the absence or presence of shikonin or CsA for 4 days. Cell division was measured via FACS analysis. **(B)** The supernatant above was measured for cytokine production via ELISA. Shikonin suppressed IFN-γ and IL-17A production but increased IL-10 and TGF-β1 levels. Data of column graphs are presented as means ± SD (*P < 0.05 and **P < 0.01, *n* = 4 wells). One of three separate experiments is shown. **(C)** Potential cytotoxicity of shikonin on T cells was measured based on cell viability using CCK-8 assays. Data are presented as means ± SD from three independent experiments (*P < 0.05 and **P < 0.01, *n* = 4 wells).

Given that shikonin significantly prolonged skin allograft survival, we then asked whether it would suppress T cell proliferation and alter cytokine secretion *in vitro*. FACS-sorted CD3^+^ T cells from naïve C57BL/6 mice labeled with CFSE were co-stimulated with anti-CD3/CD28 Abs and IL-2 in the absence or presence of shikonin or CsA for 4 days. Cell division was measured via FACS analysis. As shown in Figure 7A, shikonin, at both low and high concentrations, significantly suppressed T cell proliferation. As expected, CsA did the same. On the other hand, shikonin moderately promoted IL-10 secretion in the supernatant but dramatically inhibited production of IFN-γ (Figure 7B). Interestingly, CsA did not significantly alter IL-10 secretion although it largely blocked IFN-γ production. In contrast, shikonin at a high, but not low, concentration significantly reduced IL-17A level compared with control group. Further, CsA or high concentration of shikonin increased TGF-β1 level. These findings suggest that shikonin suppresses T cell proliferation and their production of IFN-γ and IL-17A while increasing IL-10 and TGF-β1 levels *in vitro*. To rule out the possibility that suppressive effects of shikonin on T cells were caused by its cytotoxicity, we measured cell viability using CCK-8 assays. As shown in Figure 7C, cell viability was not compromised when up to 1.0 μM shikonin was used in the cell culture, suggesting that suppression of T cell proliferation and activation by shikonin was not attributed to its cytotoxicity.

### Shikonin Suppresses mRNA Expressions of Proinflammatory Cytokines but Increases IDO and FoxP3 Gene Expressions in Skin Allografts

**Figure 8:**
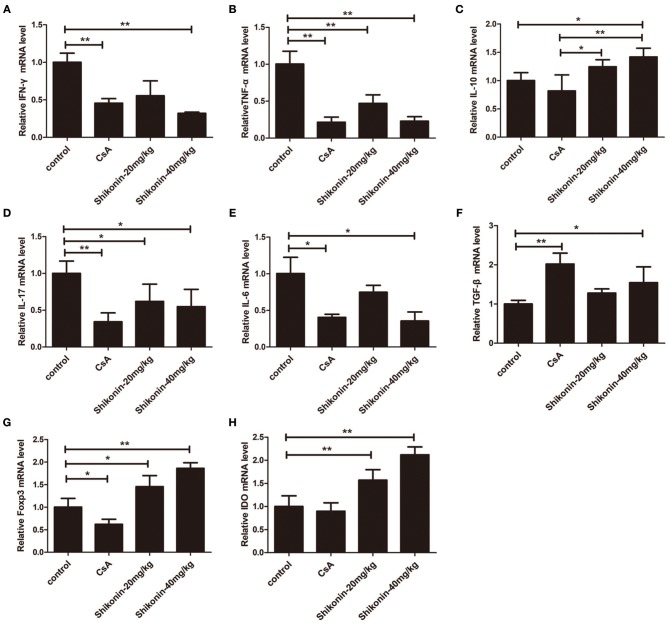
Shikonin suppresses the mRNA expressions of proinflammatory cytokines while increasing IL-10 expression in skin allografts. The mRNA levels of IFN-γ **(A)**, TNF-α **(B)**, IL-10 **(C)**, IL-17A **(D)**, IL-6 **(E)**,TGF-β1 **(F)**, FoxP3 **(G)**, and IDO **(H)** in skin allografts were measured by RT-PCR 10 days after skin transplantation and treatment with shikonin or CsA. Data of column graphs are presented as means ± SD (*P < 0.05 and **P < 0.01, *N* = 4–5 mice). One of three separate experiments is shown.

To further examine the anti-inflammatory effects of shikonin, mRNA expressions of IFN-γ, TNF-α, IL-6, IL-10, IL-17A, FoxP3, IDO, and TGF-β1 in skin allografts were determined by RT-PCR 10 days after skin transplantation as well as related treatments. As shown in Figure 8, the mRNA expressions of these proinflammatory cytokines in skin allografts were significantly downregulated after the treatment with either CsA or shikonin, especially at high doses, whereas shikonin, but not CsA, upregulated IL-10 and FoxP3 mRNA levels compared with control group. On the other hand, both CsA and high doses of shikonin augmented TGF-β1 gene expression while either low or high doses of shikonin, but not CsA, increased IDO gene expression. These findings indicate that shikonin upregulates gene expressions of IDO and FoxP3 as well as immunosuppressive cytokines IL-10 and TGF-β1 in allografts and is indeed anti-inflammatory in the context of alloimmunity.

### Shikonin Inhibits mTOR Signaling in T Cells

**Figure 9:**
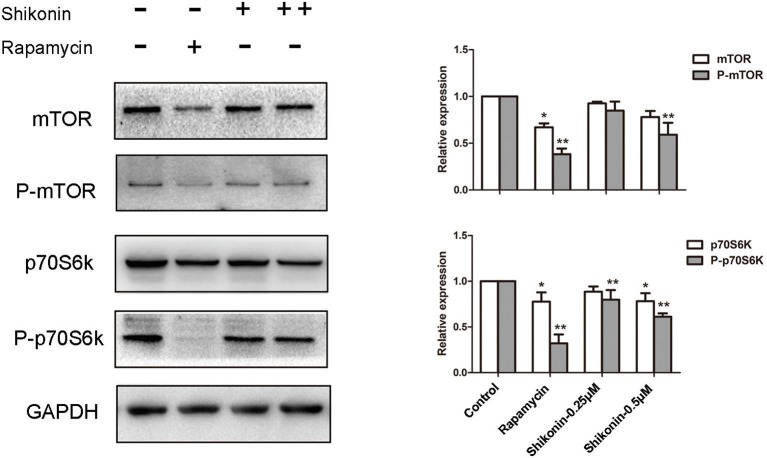
Shikonin inhibits T-cell mTOR signaling *in vitro*. The protein expressions of phosphorylated p70S6K (P-p70S6K), p70S6K, P-mTOR, and mTOR in T cells were measured using western blotting analysis 2 days after T cells were stimulated *in vitro* with anti-CD3/CD28 Abs in the absence or presence of shikonin. A representative of Western blot images of P-p70S6K, p70S6K, p- mTOR, and mTOR expressions was shown (left panel). GAPDH was used as a loading or internal control. OD values (relative to GAPDH, right panel) in column graphs are presented as means ± SD from three separate experiments (*P < 0.05 and **P < 0.01, *n* = 3).

The mTOR signaling is closely associated with the generation of Tregs since rapamycin, an mTOR inhibitor, has been shown to induce CD4^+^Foxp3^+^ Tregs (21). Given that shikonin induced CD4^+^Foxp3^+^ Tregs *in vivo and vitro*, we determined whether shikonin would have an effect on T-cell mTOR signaling. In our study, the expressions of phosphorylated p70S6K (P-p70S6K), p70S6K, P-mTOR, and mTOR proteins in T cells were measured using western blotting analysis 2 days after T cells were stimulated *in vitro* with anti-CD3/CD28 Abs in the absence or presence of shikonin. As shown in Figure 9, shikonin significantly inhibited the expression of phospho-p70S6K (both low and high concentrations: *p* < 0.01) and phospho-mTOR (high concentration: *p* < 0.01) compared to the control group. As a positive control, rapamycin largely blocked expressions of both phospho-p70S6K and phospho-mTOR. These findings indicate that induction of Tregs by shikonin is likely due to its blockade of mTOR signaling pathway.

## Discussion

An allograft is always rejected by a recipient in the absence of any treatment with a conventional immunosuppressant. However, continuously global immunosuppression usually causes side effects, including nephrotoxicity, tumors and infections. Therefore, it's necessary to seek a natural product or ingredient with potentially less side effects. Shikonin, an ingredient originally extracted from a traditional Chinese herb, has been shown to regulate immune responses and ameliorate inflammatory diseases (10, 12, 13). However, it's unknown whether shikonin has a significant impact on alloimmunity or allograft rejection. In this study, we have provided the first evidence that shikonin prolongs allograft survival in a murine model of skin allotransplantation and suppresses T cell proliferation and mTOR signaling while inducing CD4^+^Foxp3^+^ Tregs and upregulating IDO.

Tregs play an important role in maintaining immunological homeostasis and tolerance (18–20). A small subset of CD4^+^ T cells expressing the α chain of IL-2 receptor (CD25) and specifically fork head family transcription factor 3 (Foxp3) are typical Tregs (22, 23). Either induction of endogenous CD4^+^CD25^+^ Tregs or adoptive transfer of exogenous Tregs prevents autoimmune diseases and allograft rejection in many animal models (24–26). Deletion of the transcription factor Foxp3 (23, 27) or the growth factor IL-2 (28) diminishes the development or function of CD4^+^CD25^+^ Tregs. Our results showed that shikonin significantly increased the frequencies of CD4^+^Foxp3^+^ Tregs in recipients compared with control while CsA did the opposite, suggesting that shikonin and CsA suppress allograft rejection via totally differential mechanisms. As a conventional immunosuppressive drug, CsA also suppresses allograft rejection by inhibiting T cell activation (3). Interestingly, it has been shown that CsA, a widely used conventional immunosuppressant, prevents tolerance induction by costimulatory blockade (29–31) since it inhibits the expression of IL-2 (32–34), an important growth factor for Treg development (28, 35, 36). Perhaps, this is why CsA did not promote Treg generation in our study although it increased TGF-β1 expression. Therefore, shikonin rather than CsA may be used to induce allograft tolerance in futuristic study.

IDO has been shown to play an important role in Treg generation and function. DC-derived IDO is essential for Treg generation (37), expansion (38), and activation (39). IDO also suppresses allograft rejection by inducing Tregs (40, 41). Moreover, IDO-expressing MDSCs inhibits islet xenograft rejection (42) while DCs expressing IDO also suppresses allograft rejection by expanding Tregs (43). Here we found that shikonin treatment increased IDO expression in skin allografts and DCs *in vitro* and induced Tregs as well. Therefore, it's possible that shikonin promotes Treg generation via upregulating IDO expression in DCs. On the other hand, we demonstrated that shikonin hindered DC maturation post-transplantation. Thus, it's also possible that shikonin induces Tregs via suppression of DC maturation.

In present study, FACS-sorted CD4^+^CD25^−^ T cells were stimulated with anti-CD3/CD28 Ab and recombinant IL-2 or TGF-β1 in the absence or presence of shikonin for 4 days. Our data demonstrated that shikonin promoted CD4^+^Foxp3^+^ Treg differentiation nearly as effectively as TGF-β1 *in vitro*. Previous studies have shown that TGF-β1 dramatically promotes naïve CD4^+^ T cell differentiation into Tregs (44). Thus, shikonin appears to be a potent inducer of Tregs. Additionally, depleting CD25^+^ Tregs mostly reversed skin allograft survival prolonged by shikonin, suggesting that shikonin extends allograft survival likely through induction of CD4^+^CD25^+^ Tregs.

T cells play a key role in the initiation of immune responses (45). Autoreactive T cells can induce autoimmune diseases while alloreactive T cells cause graft rejection (46, 47). T cells activated in draining lymph nodes migrate to a transplanted organ/tissue and orchestrate the process of graft rejection. Then activated effector T cells produce large amounts of proinflammatory cytokines, leading to the tissue destruction and ultimate allograft rejection. Therefore, it is imperative to inhibit T cell activation in order to suppress allograft rejection. In our studies, we found that shikonin significantly suppressed the proliferation of T cells and their production of IFN-γ and IL-17A *in vitro* while increasing IL-10 and TGF-β1 levels. Further, shikonin inhibited gene expressions of proinflammatory cytokines, including IFN-γ, TNF-α, IL-6, and IL-17, in skin allografts. Thus, shikonin inhibits allograft rejection by suppressing T cell activation and expressions of proinflammatory cytokines in an allograft.

Rapamycin, an inhibitor of mTOR signaling, suppresses T cell proliferation by preventing cells from entering G1-phase (48, 49). It has been shown that rapamycin can promote CD4^+^Foxp3^+^ Treg generation (50–52). In present study, we demonstrated that shikonin induced CD4^+^Foxp3^+^ Tregs *in vivo and in vitro* and that depletion of the Tregs reversed allograft survival induced by shikonin. We then asked whether shikonin induced Tregs also by blocking mTOR signaling pathway. Indeed, shikonin effectively inhibited the protein expression of phospho-p70S6K and phospho-mTOR in T cells. Therefore, shikonin turns out to be a novel mTOR inhibitor.

In conclusion, shikonin significantly prolonged skin allograft survival in a mouse model of skin allotransplantation by promoting CD4^+^Foxp3^+^ Treg differentiation. It also suppressed T cell proliferation *in vitro* while reducing their mTOR signaling. Furthermore, shikonin inhibited expressions of proinflammatory cytokines while increasing expression of immunosuppressive cytokines and IDO in skin allografts. Our data revealed a novel role of shikonin in inducing Tregs/IDO and suppressing allograft rejection. These findings indicate that shikonin, an originally natural product, may be utilized as a potent immunosuppressive drug for the prevention of human transplant rejection in the future.

## Data Availability

The datasets generated for this study are available on request to the corresponding author.

## Author Contributions

QiZ performed experiments and wrote the manuscript. FQ and YC performed some experiments. CL, HL, and C-LL analyzed the data. QuZ and ZD edited the manuscript.

### Conflict of Interest Statement

The authors declare that the research was conducted in the absence of any commercial or financial relationships that could be construed as a potential conflict of interest.
